# Disulfide Bond Engineering of Soluble ACE2 for Thermal Stability Enhancement

**DOI:** 10.3390/ijms25189919

**Published:** 2024-09-14

**Authors:** Yoon Soo Kim, Myeongbin Kim, Hye Min Park, Hyun Jin Kim, Seong Eon Ryu

**Affiliations:** Department of Bioengineering, College of Engineering, Hanyang University, Seoul 04673, Republic of Korea

**Keywords:** soluble ACE2, thermal stability enhancement, disulfide bond, SARS-CoV-2, variant-independent therapeutics

## Abstract

Although the primary pandemic of SARS-CoV-2 is over, there are concerns about the resurgence of the next wave of related viruses, including a wide range of variant viruses. The soluble ACE2 (sACE2) inhibits the SARS-CoV-2 spike protein ACE2 interaction and has potential as a variant-independent therapeutic against SARS-CoV-2. Here, we introduce novel disulfide bonds in the wild-type sACE2-Fc by structure-guided mutagenesis, aiming to improve its stability. The stability of each mutant was assessed by a thermal shift assay to screen mutants with increased thermal stability. As a result, we identified a mutant sACE2-Fc with a significantly increased melting temperature. X-ray crystal structure determination of the sACE2 mutant confirmed the correct formation of the designed disulfide bond, and there were no significant structural disturbances. We also proved that the thermostable sACE2-Fc preserved the spike protein binding affinity comparable to the wild-type sACE2-Fc in both molecular and cellular environments, suggesting its therapeutic potential.

## 1. Introduction

Severe acute respiratory syndrome coronavirus 2 (SARS-CoV-2) infects the host cells by binding to angiotensin-converting enzyme 2 (ACE2), and the infection is responsible for the global pandemic of COVID-19 [1]. ACE2 is a membrane-bound protein widely distributed in various tissues and organs, including the heart, lungs, kidneys, and the gastrointestinal tract [2]. It is a regulator in the renin–angiotensin–aldosterone system (RAAS). It counterbalances the effects of ACE by converting angiotensin II into angiotensin-(1-7), which regulates blood pressure and maintains cardiovascular homeostasis [3,4]. The full-length ACE2 comprises 805 amino acids and consists of an extracellular domain, a transmembrane-spanning domain, and a cytosolic domain. The extracellular domain contains ACE2’s enzyme activity and serves as a receptor for SARS-CoV-2 [5,6]. 

The spike protein of SARS-CoV-2 consists of 1270 amino acids, including S1 and S2 subunits. The S1 subunit binds to ACE2 to enter the host cells, and the S2 subunit facilitates the membrane fusion [7]. The spike protein-ACE2 binding is the crucial step in viral infection [8]. Recent studies have demonstrated that a soluble ACE2 protein (sACE2) impedes the spike protein–ACE2 binding, subsequently blocking viral entry into the host cell [5,6,9,10,11,12,13,14,15,16,17,18,19,20]. sACE2 can inhibit infection from SARS-CoV-2 or related viruses with less variant dependency [21]. Although the primary pandemic of SARS-CoV-2 is over, there are concerns about the resurgence of a next wave of related viruses [22,23,24]. To deal with that situation, the soluble receptor approach can be a good choice for a therapeutic able to inhibit variant viruses. The full-length sACE2 exhibits a short half-life of 8 h in vivo, presenting challenges for therapeutic applications [25]. Approaches with domain-engineering [5], directed evolution [26], high-affinity engineering [27], and the introduction of fusion proteins [5,6] have improved the in vivo stability and activity of sACE2. 

Previous studies on sACE2 improvement mainly exploited protein domain fusion, random mutagenesis, or affinity engineering. Thus, more rational approaches for structural stabilization using three-dimensional structural information would result in sACE2 molecules that stabilize the protein domain without structural perturbation. In this study, we introduced structure-based novel disulfide bonds into sACE2-Fc, showing that the appropriately introduced disulfide bonds enhanced the melting temperature significantly without structural perturbation. Based on the structure of native sACE2 [28], we identified potential disulfide bond sites that would increase the protein stability without affecting its structural integrity. For the stability screening of sACE2-Fc mutants, we used a thermal shift assay (TSA) to measure the melting temperatures of each mutant. Then, we conducted crystallization using the mutant with the highest melting temperature. The crystal structure determination verified the formation of a disulfide bond in the structure. The competitive binding assay also assessed the binding affinity to the SARS-CoV-2 spike protein. The stabilized sACE2-Fc has the potential as a novel therapeutic against SARS-CoV-2 and related viruses.

## 2. Results and Discussion

### 2.1. Design of Stabilizing Disulfide Bonds

**Figure 1:**
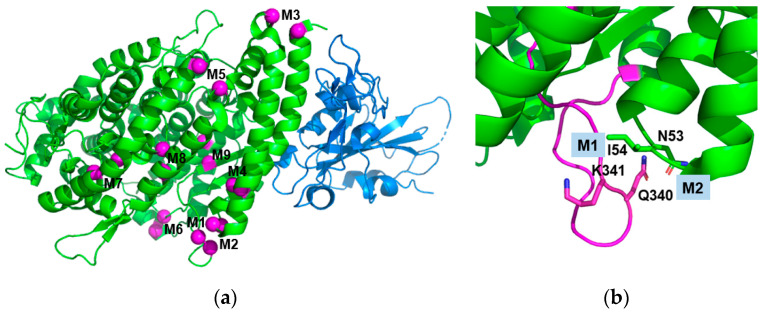
Design of novel disulfide bonds. (**a**) Selected residue pairs for novel disulfide bonds are presented as balls in magenta. The figure is drawn from the sACE2:SARS-CoV-2 spike (RBD) complex structure (PDB ID: 6VW1) [31]. The sACE2 and SARS-CoV-2 spike proteins are in green and blue, respectively. The nine disulfide bond pairs in Table 1 (M1–M9) are indicated on the pairs of the magenta balls. (**b**) The close-up view of the loop 331–347 region contains the M1 and M2 disulfide bond pairs. The figure is drawn from PDB ID: 1R42 and is in the same view as (**a**), and loop 331–347 is in magenta. The residues mutated for the M1 (N53C/Q340C) and M2 (I54C/K341C) disulfide bonds are presented as sticks.

**Figure 2:**
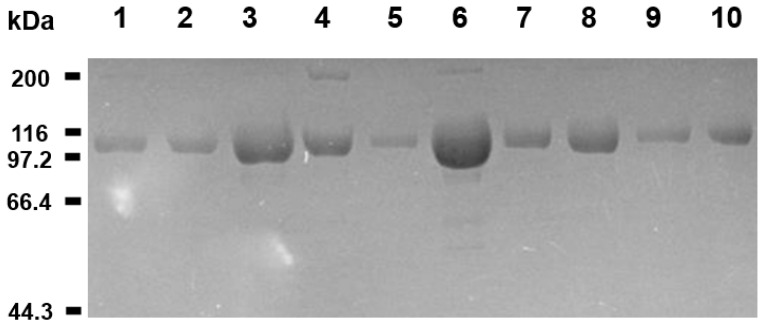
Purified sACE2-Fc proteins. The Coomassie blue-stained SDS-PAGE gel image is presented for the purified sACE2-Fc proteins. The sACE2-Fc proteins are attached to Fc for expression and purification (see Section 3 Methods). The minor bands in the 200 kDa region are likely the dimer of sACE2-Fc fusion that remains in the sample due to incomplete reduction of the disulfide bond-linked original sACE2-Fc dimer. All bands in the SDS-PAGE gel contain Fc because the samples were purified by the protein A affinity chromatography, and the sizes of the bands indicate that they are sACE2-Fc fusion proteins. The SDS-PAGE gel is shown here to verify the purity of the samples. The spike protein binding activity of the samples was verified in the molecular and cellular assays (see the Section 2.4 and Section 2.5). Lane 1 represents the wild-type protein, and lanes 2–10 are for the mutant sACE2-Fc proteins (M1–M9) in Table 1. Positions for the molecular weight markers are indicated.

The amino acid candidates for substitution with cysteine were selected based on the three-dimensional structure of native sACE2 (PDB ID 1R42) [28] (Figure 1 and Table 1). We chose nine pairs of disulfide bond candidates presented in Table 1 and named M1–M9. In the selection, we employed two principles. First, the C-alpha distance of the selected residue pair was within the range of 4.5 to 7.0 Å, considering the average C-alpha distance of disulfide bonds in natural proteins is 5.6 Å [29]. Second, we preferred the residue pairs whose beta-carbon directions are toward the partnering residue to enable the formation of disulfide bonds without much strain on the main chain structure. We selected the residue pairs that could stabilize specific loops, helix–helix interactions, and strand–helix interactions that contribute to the protein folding stability. We also chose the candidate pairs not close to the binding sites for the SARS-CoV-2 spike protein. By inspecting the structure of the extracellular region of ACE2, we found that the loop 331–347 region has a long and extended structure (Figure 1b), and we selected two pairs of disulfide bonds (M1 and M2) to stabilize the loop region. M3 and M9 were to stabilize helix–loop and helix–strand interactions, respectively. M4–M8 were chosen to stabilize helix–helix interactions. To minimize sACE2’s intervention in the physiological blood pressure modulation, we introduced inactivating mutations (the H374N and H378N mutations) [30]. The sACE2 (residues 1–615) with catalytically inactivating mutations was used to engineer disulfide bonds. We expressed sACE2 proteins as attached to Fc because the expression yield of sACE2 was not good, and the Fc fusion would increase the serum retention time. While the Fc-fusion would increase the serum retention time of sACE2, the stabilizing disulfide bond would extend the half-life of the functional sACE2. All mutated sACE2-Fc fusion proteins were purified to a high purity (Figure 2).

### 2.2. Screening for the Rmo-Stable Mutants

**Figure 3:**
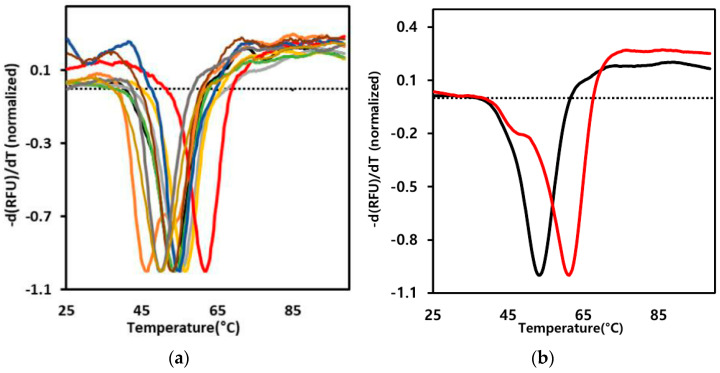
Melting curves by the thermal shift assay. The first derivative melting curves of the wild-type and mutant sACE2-Fc proteins are presented. The Tm experiments were carried out with sACE2-Fc fusion proteins. Because we observed one melting transition point, the unfoldings of sACE2 and Fc domains are likely correlated. The dotted line represents the –d(RFU)/dT value at 25 °C of the wild type. (**a**) The melting curves of the wild type (black), the M2 mutant (red), and the other mutants in Table 1 (M1: yellow, M3: gold, M4: violet, M5: green, M6: grey, M7: brown, M8: blue, and M9: olive) are presented together. We performed three independent experiments, and the Tm results were similar. (**b**) The melting curves of only the wild type (black) and the M2 mutant (red) are presented for comparison.

To estimate the effect of a disulfide bond formation on sACE2, we measured the denaturation temperature of the protein by using the thermal shift assay (TSA) (Figure 3). In this assay, we used sACE2 attached to human Fc. Each mutant’s melting temperature (Tm) was estimated using the melting curves. The right-shifted graph (red line) in Figure 3, which was the I54C/K341C mutant (M2), exhibited the most significant increase in the melting temperature. Other mutants showed melting curves similar to the wild type (black line) or left-shifted graphs for lower-temperature melting. The lower-temperature melting mutants likely have structural/protein folding disturbances due to the disulfide introduction. Table 1 shows the observed melting temperatures of the wild-type and mutant proteins. The melting temperature of M2 was 61.2 °C, indicating a significant increase of 8.1 degrees compared to the wild type. The increased melting temperature is an indication of the protein domain stability. Even though the melting temperature is much above the physiological temperature, the increased protein domain stability can prohibit the domain from a partial or global unfolding at the physiological temperature. The stabilization likely extends the half-life of the functional state of sACE2, enhancing its therapeutic effects. 

In the structure of the loop 331–347 region (Figure 1b), the side chain of Ile 54 points toward the loop, and the disulfide bond formation between Ile 54 and Lys 341 would strengthen the interactions between loop 331–347 and the Ile 54-containing loop. Interestingly, the disulfide bond between Asn 53 and Gln 340 (M1) adjacent to M2 improved the melting temperature by 3.5 degrees less than that of M2 (8.1 degrees). The side chains of both Asn 53 and Gln 340 point outside the protein fold, indicating no direct interactions mediated by the side chains of the two residues. Thus, disulfide bond formation between the two residues does not have a strong effect on the stabilization of interacting residues, and the stabilizing effect of the flexible loop 331–347 would be relatively small compared to M2. The disulfide bond formation between residues in helices or between residues in loop/strand and helix did not improve Tm, and in some cases, Tm was decreased (Figure 3a and Table 1). 

### 2.3. Structure Determination of the Thermostable Mutant

**Figure 4:**
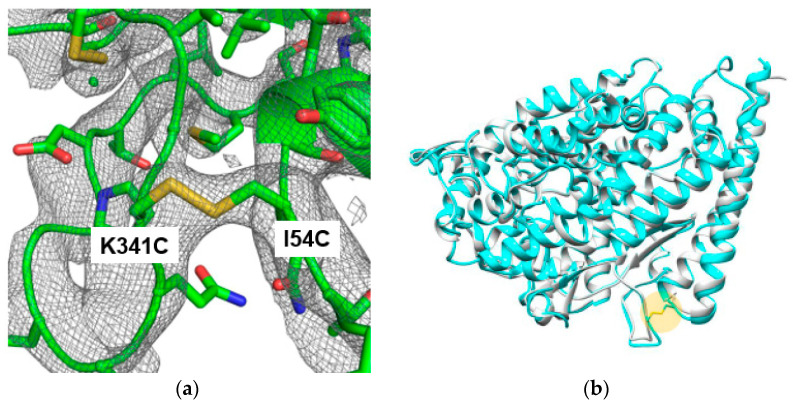
Structural analysis of the M2 mutant sACE2. (**a**) The 2mFo-DFc map of the sACE2 mutant. The map is contoured at 0.7σ. In the protein model, the oxygen, nitrogen, and sulfur atoms are in red, blue, and yellow, respectively. The carbon atoms are in green. (**b**) Structural superposition of the wild-type (PDB ID: 1R42) (gray) and mutant (cyan) sACE2 proteins. The average Cα RMSD value is 0.58 Å. The Cys54–Cys341 disulfide bond is indicated as a yellow circle.

The crystal structure of the most thermostable sACE2 mutant (M2) was determined to verify the formation of the designed disulfide bond without undesirable structure changes. For crystallization, the Fc tag was removed from the sACE2-Fc fusion protein to eliminate potential interference with crystal packing and to obtain high-quality crystals. The Fc-removed and purified M2 protein was analyzed with gel filtration chromatography to verify the protein homogeneity as a monomer in solution. The crystal belonged to the space group H3. The unit dimensions were a = 180.32 Å, b = 180.32 Å, c = 69.49 Å, α = β = 90°, and γ = 120° (Table 2). The crystal structure was determined by the molecular replacement method using the structure of native sACE2 (PDB ID: 1R42) as the target. The resulting electron density showed clear density for a new disulfide bond between two mutated cysteines (Figure 4a). Due to the limited resolution of 3.5 Å, there were missing densities for side chain regions of the long side chain residues such as glutamine and aspartate. However, main-chain densities were well connected, and disulfide bond density was well defined. 

Structural comparison of the mutant with the wild type demonstrated high similarity with a root mean squared deviation (RMSD) of 0.58 Å for Cα atoms. There were no regions with significant structural deviations (Figure 4b). The structural similarity of the stable M2 mutant with the wild type indicated that the mutant likely does not affect ACE2’s functions. The disulfide bond formation can decrease transient thermal motions in flexible regions of the protein structure, resulting in a Tm increase [32,33]. In addition to its antiviral applications, sACE2 has a therapeutic potential for angiotensin II-dependent hypertension [25]. In this case, the similarity of the native structure in M2 would be beneficial, too. 

### 2.4. Binding to the Spike Protein

To characterize the binding affinity of the sACE2 proteins to the SARS-CoV-2 spike protein, we used the surface plasmon resonance (SPR) technique. The wild-type or M2 mutant sACE2-Fc proteins were injected as an analyte over the immobilized spike protein at an increasing concentration (Figure S1). The kinetic analysis indicated that both the wild-type (K_D_ = 23.2 nM) and the mutant (K_D_ = 22.1 nM) displayed a similar affinity to the spike protein (Figure S1). The dissociation constants were comparable to the previously reported values where the spike protein exhibited a dissociation constant of approximately 20–40 nM for binding with sACE2-Fc [34]. Interestingly, M2 exhibited lower K_on_ and K_off_ compared to those of the wild type (1.70 × 10^4^ vs. 8.48 × 10^4^ for K_on_ and 3.75 × 10^−4^ vs. 1.97 × 10^−3^ for K_off_). Because both K_on_ and K_off_ are low, the K_D_ of M2 (22.1) was similar to that of the wild type (23.2). Currently, it is not clear how the loop stabilization in M2 affected the kinetic behavior of sACE2-Fc. However, the different kinetic properties may reflect the propagation of the loop stabilization to the spike-binding helix stability (Figure 1b) through a long-range effect of protein conformation [35]. In this case, the effect is on both K_on_ and K_off_, compensating each other to minimize the K_D_ difference.

### 2.5. Cell-Based Competition Analysis

**Figure 5:**
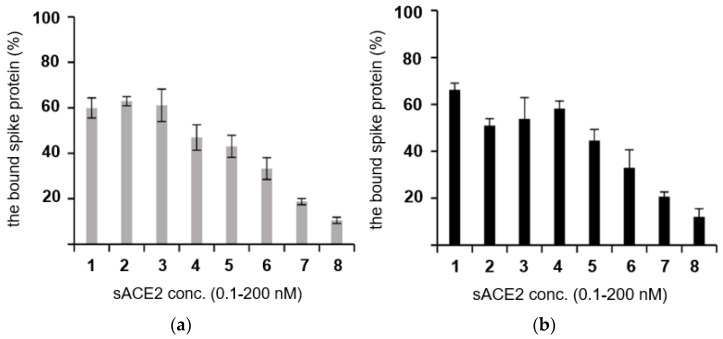
Competitive binding assay in cells. The binding affinity of the sACE2-Fc’s in a cellular condition was estimated by a competitive assay. The wild-type (**a**) and the M2 mutant (**b**) sACE2-Fcs were added to the spike protein/ACE2 binding assay (see the text). The Y-axis represents the percentage of the spike protein bound to the surface ACE2 on the cell. The X-axis represents different concentrations of sACE2-Fc used in the assay. Bars 1–8 in (**a**) and (**b**) represent 0.1, 0.3, 0.8, 2.5, 7.4, 22.2, 66.7, and 200 nM of the wild-type (**a**) and the mutant sACE2-Fc (**b**) proteins, respectively. The amount of the spike protein bound to sACE2-Fc decreased in a dose-dependent manner. The assay was triplicated, and the standard deviation is indicated in each bar. In the assay, we prepared the 200 nM protein solution and serial-diluted it three-fold each time. Thus, the lowest concentration was 0.1 nM (bar 1), not 0.0.

The binding of the sACE2 mutant to the SARS-CoV-2 spike protein in the cellular environment was analyzed by a competitive binding assay using a cell-based enzyme-linked immunosorbent assay (Figure 5). For the competitive binding assay, the wild-type and M2 sACE2-Fc proteins were treated with the spike protein on the ACE2-overexpressing HEK293 cells. The sACE2-Fc proteins competed with the ACE2 receptor expressed on the cell membrane for interaction with the spike protein. The spike protein bound to the cell-surface ACE2 decreased as the concentration of the sACE2-Fc proteins increased. In Figure 5, the spike protein bound to the cell-surface ACE2 decreases similarly in both the wild-type and M2 sACE2-Fc. The result demonstrated that the wild-type and M2 sACE2-Fc proteins bind to the spike protein with similar affinity, thereby preventing the spike protein from binding to the ACE2 expressed on the cell surface. Thus, the Cys54–Cys341 disulfide bond in M2 appears to have a minimal impact on the ACE2’s binding to the spike protein.

## 3. Materials and Methods

### 3.1. Cloning, Expression, and Purification of Proteins

The plasmid encoding for the extracellular region of human ACE2 (residues 1–615, UnitProt ID: Q9BYF1) was subcloned into pcDNA™3.1/myc-His A plasmid vector (Invitrogen, Waltham, MA, USA) containing human IgG Fc region and 6-histidine tag using KpnI and BamHI as restriction sites. Mutations were generated using the QuikChange Site-Directed Mutagenesis (Stratagene, Santa Clara, CA, USA) protocol. In addition, a catalytically inactivated ACE2 (the H374N and H378N mutations) was generated. The receptor binding domain (RBD) (residues 319–541, UniProt ID: P0DTC2) of the SARS-CoV-2 spike protein was subcloned into the same vector as mentioned above. The full-length ACE2 was subcloned into pcDNA™3.1/myc-His. All proteins were expressed in ExpiCHO cells. After thawing, the cells were cultured in ExpiCHO Expression Medium (Thermo Fisher Scientific, Waltham, MA, USA) up to 6 × 10^6^ cells/mL in a humidified shaking incubator at 37 °C, with 120 rpm and 8% CO_2_. The cells were split to 3–4 × 10^6^ cells/mL, and when the cell density reached 1 × 10^7^ cells/mL, they were diluted to 6 × 10^6^ cells/mL by ExpiCHO Expression Medium appropriate for transfection. Transfections were carried out in 125 mL Erlenmeyer flasks with filtered cap (SPL Life Sciences, Pochon, Republic of Korea), using ExpiCHO Expression System Kit (Thermo Fisher Scientific, Seoul, Republic of Korea) according to the manufacturer’s protocol. For transfection on a 25 mL scale, ExpiFectamine™ CHO transfection reagent and plasmid DNA in the range of 0.5–1.0 µg/mL were diluted with OptiPRO SFM (Thermo Fisher Scientific, Seoul, Republic of Korea). Plasmid DNA and diluted ExpiFectamine™ CHO reagent were mixed and incubated for 1–2 min. The mixed ExpiFectamine™ CHO reagent and plasmid DNA were slowly added to the cultured cells. Next, 6 mL of ExpiCHO™ Feed and 150 µL of ExpiCHO™ Enhancer were added to transfected cells 18–22 h post transfection; then, cells were transferred to a humidified 5% CO_2_ incubator at 32 °C with 120 rpm. After 10–11 days, the supernatant was harvested, and the protein was purified with a typical yield of 0.1 mg/mL.

All purification procedures were performed at 4 °C. Cells were harvested 10–12 days post transfection, and the culture solutions were centrifuged at 6000 rpm for 20 min. The supernatant was filtered through 0.45 µm nitrocellulose mixed ester (MCE) membrane filters (Advantec, Tokyo, Japan). For purification of the spike RBD protein with hexa-histidine tag, the TALON™ column (Qiagen, Hilden, Germany) was used. The column resin was regenerated by 200 mM EDTA pH 7.5, 50 mM Cobalt (Il) chloride, and 300 mM NaCl in turn and equilibrated with an equilibration buffer of 50 mM Tris-HCI pH 7.5 and 200 mM NaCl before protein binding. After the protein binding from the filtered culture media onto the TALON™ column, it was washed with 120 mL of the washing buffer (50 mM Tris-HCI pH 7.5 and 1 M NaCl). The washing procedure was repeated to purify high-purity proteins with 70 mL of 50 mM Tris-HCI pH 7.5, 500 mM NaCl, and 25 mM imidazole. After two washings, the protein was eluted with an elution buffer (50 mM Tris-HCI pH 7.5, 500 mM NaCl, and 500 mM imidazole). Lastly, the protein was desalted with PBS buffer using the HiTrap™ Desalting column (Cytiva, Marlborough, MA, USA). For the purification of Fc-tagged sACE2 proteins, a protein A column (Qiagen) was used. The column resin was regenerated with an elution buffer (50 mM glycine pH 3.5) and equilibrated with an equilibration buffer (50 mM Tris-HCI pH 7.5 and 200 mM NaCl) before protein binding. After the protein binding, the column was washed with 100 mL of the equilibration buffer. The protein was eluted with an elution buffer (50 mM glycine pH 3.5) and then desalted with TBS buffer (20 mM Tris-HCl pH 7.5 and 150 mM NaCl). The desalted protein was concentrated to 1 mg/mL and stored at −70 °C.

### 3.2. Thermal Shift Assay

Thermal shift assay was performed to measure the melting temperature (Tm) of the wild-type and mutant sACE2-Fc proteins using the Protein Thermal Shift™ Dye Kit (Thermo Fisher Scientific, Seoul, Republic of Korea). The total reaction volume per well was 20 µL, including 10 µL of 0.5 mg/mL protein, 8 µL of TBS buffer, and 2 µL of Protein Thermal Shift™ Dye (Applied Biosystems, Foster City, CA, USA) diluted to 10× in TBS buffer before use. The samples were heated in the PCR system from 25 to 90 °C and the fluorescence was measured. For real-time fluorescence measurement, the Applied Biosystems 7500 Real-Time PCR System (Thermo Fisher Scientific, Seoul, Republic of Korea) was used, and the melting curve was obtained using the melting curve protocol in the Protein Thermal Shift^TM^ software v1.4 (Thermo Fisher Scientific, Seoul, Republic of Korea).

### 3.3. Crystallization and Structure Determination

The Fc tag was removed from the sACE2-Fc mutant using the QuikChange Site-Directed Mutagenesis (Stratagene). Protein expression was carried out as described above, and the secreted protein in the culture medium was purified using the TALON™ column (Qiagen). Eluted protein was further purified by the gel filtration chromatography using Superdex 200 Increase 10/30 GL (GE Healthcare, Chicago, IL, USA) in TBS buffer (20 mM Tris-HCl pH 7.5 and 150 mM NaCl). Finally, the protein was concentrated to 20 mg/mL using Amicon^®^ Ultra-15 centrifugal filter (10 kDa cutoff, Merck, Darmstadt, Germany), and aliquots were stored at −70 °C. Crystal screening was performed at 18 °C using the sitting drop vapor diffusion method in drops containing 300 nl of the protein and reservoir solution. The drops were equilibrated against 50 µL of reservoir solutions. Initial trials were performed using commercial crystal screening kits (Crystal Screen, Hampton Research, Aliso Viejo, CA, USA). The crystallization solution for the optimal crystals consisted of 0.1 M HEPES pH 7.5, 20% PEG 10,000, and Tris pH 7.5.

Diffraction data were collected at Pohang Accelerator Laboratory (PAL) beamline 7A. The crystals were flash-frozen in nitrogen gas. The 20% glycerol was added to the crystals to protect them during freezing. The X-ray diffraction data collected at 3.5 Å resolutions were processed with the program HKL2000 [36]. The crystals belonged to the space group H3. The crystal structure was determined by the molecular replacement using the structure of ACE2 (PDB ID: 1R42). The subsequent refinement was carried out by using the programs Coot [37] and Phenix refine [38]. The data collection and refinement statistics are presented in Table 2.

### 3.4. Binding Affinity and Kinetic Analysis by SPR

The binding affinity and kinetics of the sACE2-Fc proteins to the SARS-CoV-2 spike protein were measured using the iMSPR-mini SPR instrument (ICLUBIO, Ansan-si, Republic of Korea). The purified spike protein was covalently immobilized by the amine coupling method on a research-grade carboxylic acid (COOH) sensor chip (ICLUBIO) at 30 µg/mL. Then, the purified sACE2-Fc wild-type and mutant proteins were prepared by a 2-fold serial dilution in a running buffer (PBS and 0.05% Tween 20) with concentrations ranging 15.0–120 nM for the wild type and 13.8–110 nM for the mutant, respectively. Serial dilutions of the purified sACE2-Fc mutant and wild-type proteins were injected over the immobilized spike protein at an increasing concentration, with a flow rate of 20 µL/min. The association time was 460 s, followed by a dissociation time of 900 s, in a running buffer (PBS and 0.05% Tween 20). The sensor chip was regenerated with 10 mM glycine buffer pH 1.5 at the end of the last cycle at 20 µL/min for 3 min followed by 5 min of stabilization. Data were fitted to a 1:1 binding model and analyzed using the TraceDrawer™ v1.9.1 (TraceDrawer, Uppsala, Sweden) data analysis software.

### 3.5. Competitive Binding Assay by Cell-Based ELISA

Human embryonic kidney 293 cells (HEK293) were transfected with a pcDNA™ 3.1/myc-His A plasmid encoding the full-length ACE2. A competitive binding assay using the cell-based enzyme enzyme-linked immunosorbent assay was performed to measure the binding affinity of the sACE2-Fc mutant to the spike protein in the cell environment. One day after transfection, cells were washed once and fixed with 4% formaldehyde dissolved in DPBS (Welgene) for min at 26 °C. The plate was blocked with 160 µL of blocking buffer with 5% (*w*/*v*) skim milk in PBS at 26 °C for 2 h and washed three times with PBS. The spike protein was prepared at a concentration of 40 nM and diluted with 5% (*w*/*v*) skim milk in PBS pH 7.5. The wild-type and mutant sACE2-Fc proteins were prepared by three-fold serial dilutions starting from 200 nM with 5% (*w*/*v*) skim milk in PBS pH 7.5. Then, the mixtures of the spike protein with the sACE2-Fc wild-type and mutant proteins were pre-incubated for 30 min, treated in each well, and incubated for 3 h at 26 °C. After washing once with PBST containing 0.05% Tween 20 and thrice with PBS, SARS-CoV-2 S monoclonal antibody (M01) (1:1000, Abnova, Taoyuan City, Taiwan) was added to each well as a primary antibody and incubated overnight at 4 °C. The next day, after repetitive washing, the horseradish peroxidase (HRP) conjugated goat anti-mouse IgG antibody (1:2000, Genedepot, Baker, TX, USA) was added as a secondary antibody and incubated for 2 h at 26 °C. After washing each well twice with PBST and thrice with PBS, 60 µL of TMB substrate solution (KOMA BIOTECH, Seoul, Republic of Korea) was added to each well and incubated for 20 min at 37 °C. Lastly, 60 µL of the stop solution (0.18 M sulfuric acid) was added to stop the reaction, and the absorbance at 450 nm was measured using an Emax microplate reader (Molecular Devices, San Jose, CA, USA).

## 4. Conclusions

The soluble ACE2 (sACE2) has potential as a SARS-CoV-2 therapeutic. Novel disulfide bonds were designed and tested to increase the stability of sACE2-Fc, which is critical for its therapeutic use. The introduction of the new disulfide bond Cys54–Cys341 increased the thermal stability of the sACE2-Fc mutant by more than eight degrees. The crystal structure determination of the sACE2 mutant confirmed the formation of the designed disulfide bond without any significant structural changes throughout the sACE2 structure. The SPR and competitive binding assays confirmed that the sACE2-Fc mutant had spike protein binding characteristics comparable to the wild-type sACE2-Fc. Thus, the sACE2-Fc with the engineered disulfide bond has promise as a longer-lasting and effective therapeutic against SARS-CoV-2.

## Figures and Tables

**Table 1:** Melting temperature of the sACE2-Fc wild type and mutant proteins.

sACE2-Fc Mutants	Structural Rationale	Cα Distance (Å)	Tm (°C)	ΔTm (°C)
Wild type	na	na	53.1	0
N53C/Q340C (M1)	flexible loop stabilization	5.9	56.6	3.5
I54C/K341C (M2)	flexible loop stabilization	6.0	61.2	8.1
I21C/E87C (M3)	helix–loop stabilization	6.6	45.8	−7.3
M62C/S47C (M4)	Helix–helix stabilization	4.8	55.4	2.3
A193C/V107C (M5)	helix kink–helix kink stabilization	6.5	53.9	0.8
V364C/V298C (M6)	helix–helix stabilization	6.6	49.3	−3.8
S502C/R169C (M7)	helix–helix stabilization	6.2	53.5	0.4
N508C/S124C (M8)	helix–helix stabilization	5.6	54.7	1.6
A348C/H378C (M9)	helix–strand stabilization	6.3	50.1	−3.0

**Table 2:** Data collection and refinement statistics.

Resolution range (Å)	34.08–3.50 (3.63–3.50) *
Space group	H3
Unit cell a, b, c (Å) α, β, γ (°)	180.32, 180.32, 69.49 90.00, 90.00, 120.00
Total reflections	26,877 (2090)
Unique reflections	9802 (912)
Redundancy	2.7 (2.3)
Completeness (%)	92.25 (86.5)
I/σI	8.67 (4.52)
R_merge_ (%)	9.7 (20.5)
CC_1/2_	0.984 (0.885)
Reflections used in refinement	9799 (912)
Reflections used for R-free	983 (91)
R-work/R-free	20.0 (23.2)/25.9 (29.5)
Number of total atoms	4866
Protein residues	597
RMS (bonds) (Å)	0.031
RMS (angles) (°)	0.8
Ramachandran plot (%) Favored/allowed/outliers	93.95/5.88/0.17
Average B-factor (Å^2^)	62.22

* The numbers in parentheses are for the last shell.

## Data Availability

The original contributions presented in the study are included in the article/supplementary materials, further inquiries can be directed to the corresponding author/s.

## Supplementary Materials

The supporting information can be downloaded at: https://www.mdpi.com/article/10.3390/ijms25189919/s1.
