# Supplementary material for: Disulfide Bond Engineering of Soluble ACE2 for Thermal Stability Enhancement
Source: Int J Mol Sci. 2024 Sep 14;25(18):9919. doi: 10.3390/ijms25189919 (PMC11432317; doi:10.3390/ijms25189919)
Supplement: Supplementary file 1 [file ijms-25-09919-s001.zip › ijms-3177428-supplementary.pdf]

## Supplementary Information

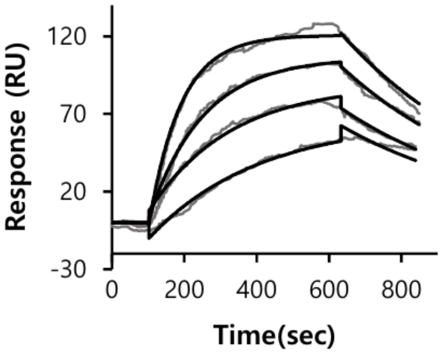


(**a**)

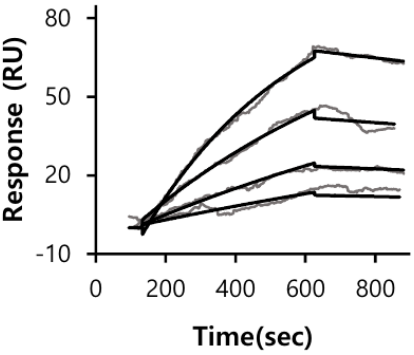


(**b**)

| | Wild type | M2 |
|---|---|---|
| $K_{on}$ (1/M s) | 8.48E+4 | 1.70E+4 |
| $K_{off}$ (1/s) | 1.97E-3 | 3.75E-4 |
| $K_D$ (nM) | 23.2 ± 0.6* | 22.1 ± 0.5* |

**Figure S1.** Binding affinity measurement by SPR. The binding affinity of sACE2-Fc's to the SARS-CoV-2 spike protein was measured by surface plasmon resonance (SPR). The sensorgrams of (a) the wild-type and (b) the M2 mutant. The wild-type and mutant proteins flew over the sensor chip as analytes at increasing concentrations. The sensor chip was pre-coated with the spike protein. Each sACE2-Fc protein was diluted to different concentrations (from 15 to 120 nM for the wild-type and from 13.8 to 110 nM for the M2 mutant) before injection. The association constant ($K_{on}$), the dissociation constant ($K_{off}$), and the equilibrium dissociation constant ($K_D$) were calculated by the 1:1 binding model using the TraceDrawer™ data analysis software. * the means ±standard deviation (SD) values. The SPR signals were relatively noisy even though we tried to optimize the SPR measurement conditions by varying flow rate and association time. The noise, indicating an unstable interaction, may come from a less-optimal coating of the spike protein on the sensor chip. Because the spike protein is relatively small compared to sACE2 (see Fig. 1), the interaction efficiency would depend on the orientation of the spike protein coated on the surface of the sensor chip. The partial interaction would cause the signal noise.
